# Synthesis and Characterization of Temperature-Responsive *N*-Cyanomethylacrylamide-Containing Diblock Copolymer Assemblies in Water

**DOI:** 10.3390/polym13244424

**Published:** 2021-12-16

**Authors:** Nicolas Audureau, Fanny Coumes, Clémence Veith, Clément Guibert, Jean-Michel Guigner, François Stoffelbach, Jutta Rieger

**Affiliations:** 1 Polymer Chemistry Team, Institut Parisien de Chimie Moléculaire (IPCM), Sorbonne Université & CNRS, UMR 8232, 4 Place Jussieu, CEDEX 05, 75252 Paris, France; nicolas.audureau@sorbonne-universite.fr (N.A.); fanny.coumes@sorbonne-universite.fr (F.C.); clemence.veith@hotmail.fr (C.V.); 2 Laboratoire de Réactivité de Surface (LRS), Sorbonne Université, CNRS, 4 Place Jussieu, CEDEX 05, 75252 Paris, France; clement.guibert@sorbonne-universite.fr; 3 Institut de Minéralogie, de Physique des Matériaux et de Cosmochimie (IMPMC)-IRD-MNHN, Sorbonne Université & CNRS, UMR 7590, CEDEX 05, 75252 Paris, France; jean-michel.guigner@sorbonne-universite.fr

**Keywords:** polymerization-induced self-assembly, RAFT polymerization, self-assembly, amphiphilic block copolymers, micelles, worms, vesicles, UCST, turbidimetry, synchrotron SAXS

## Abstract

We have previously demonstrated that poly(*N*-cyanomethylacrylamide) (PCMAm) exhibits a typical upper-critical solution temperature (UCST)-type transition, as long as the molar mass of the polymer is limited, which was made possible through the use of reversible addition-fragmentation chain transfer (RAFT) radical polymerization. In this research article, we use for the first time *N*-cyanomethylacrylamide (CMAm) in a typical aqueous dispersion polymerization conducted in the presence of poly(*N,N*-dimethylacrylamide) (PDMAm) macroRAFT agents. After assessing that well-defined PDMAm-*b*-PCMAm diblock copolymers were formed through this aqueous synthesis pathway, we characterized in depth the colloidal stability, morphology and temperature-responsiveness of the dispersions, notably using cryo-transmission electron microscopy (cryo-TEM), dynamic light scattering (DLS), small angle X-ray scattering (SAXS) and turbidimetry. The combined analyses revealed that stable nanometric spheres, worms and vesicles could be prepared when the PDMAm block was sufficiently long. Concerning the thermoresponsiveness, only diblocks with a PCMAm block of a low degree of polymerization (*DP*_n,PCMAm_ < 100) exhibited a UCST-type dissolution upon heating at low concentration. In contrast, for higher *DP*_n,PCMAm_, the diblock copolymer nano-objects did not disassemble. At sufficiently high temperatures, they rather exhibited a temperature-induced secondary aggregation of primary particles. In summary, we demonstrated that various morphologies of nano-objects could be obtained via a typical polymerization-induced self-assembly (PISA) process using PCMAm as the hydrophobic block. We believe that the development of this aqueous synthesis pathway of novel PCMAm-based thermoresponsive polymers will pave the way towards various applications, notably as thermoresponsive coatings and in the biomedical field.

## 1. Introduction 

Thermoresponsive polymers presenting an upper or a lower critical solution temperature (UCST or LCST, respectively) in water have been widely investigated, in particular for the development of biomaterials [1,2,3,4,5,6]. Over the past decade, the study of novel neutral UCST polymers, mainly based on (meth)acrylamide (co)polymers [7,8,9,10,11], broadened the library of available materials. Since 2012, the UCST-behavior of poly(acrylamide-*co*-acrylonitrile) (P(Am-*co*-AN)) [7], a neutral statistical copolymer of acrylamide and acrylonitrile, has been revealed and since then it has been largely studied for numerous applications [12,13,14,15,16]. While it had been generally synthesized by free radical polymerization in DMSO solution, Ferji et al. [17] and some of us [18] demonstrated recently that well defined P(Am-*co*-AN) copolymers can be synthesized directly in water, using the reversible addition-fragmentation chain transfer (RAFT) radical polymerization. Furthermore, we demonstrated that it was possible to synthesize in situ P(Am-*co*-AN) based block copolymer nanoparticles using the polymerization-induced self-assembly (PISA) process in water [18]. Thermoresponsive spherical particles and short worms, but surprisingly no vesicles could be prepared. In addition, the use of AN as a comonomer is challenging because it is highly volatile and it has a different reactivity compared to acrylamides [18,19]. In addition to technical and reproducibility issues, the monomer distribution in the polymer chains strongly depends on the polymerization method. Looking for a more robust and simple system, we have recently developed a novel UCST polymer platform. We demonstrated that neutral poly(*N*-cyanomethylacrylamide) (PCMAm), possessing the same function groups as P(Am-*co*-AN) but merged in a single monomer unit, exhibit a sharp and reversible UCST-type transition in water. The cloud point (T_CP_) depended on the number-average degree of polymerization (*DP*_n_) and ranged between 54 °C and 90 °C [11].

In this study, we wanted to exploit this new UCST platform as a building block to synthesize amphiphilic diblock copolymers assemblies that are possibly thermoresponsive. For that purpose, we used PISA mediated by RAFT in the presence of a neutral poly(*N*,*N*-dimethylacrylamide) macroRAFT agent used as a hydrophilic stabilizer. The individual block lengths, i.e., *DP*_n_s, of the PDMAm and PCMAm blocks were varied systematically to investigate how they influence the colloidal stability of the nanoparticles and the type of morphology obtained. As the second block is thermoresponsive, we also investigated the impact of temperature variation by means of cryo-transmission electron microscopy (cryo-TEM), dynamic light scattering (DLS), synchrotron small angle X-ray scattering (SAXS) and turbidimetry.

## 2. Materials and Methods

### 2.1. Materials

*N*-Cyanomethylacrylamide and ethyl 2-(butylthiocarbonothioylthio)propanoate (CTA-1) were synthesized according to previously described protocols [11]. 2,2′-Azobis(isobutyronitrile) (AIBN, ≥98%, Aldrich, France), 2,2′-azobis[2-(2-imidazolin-2-yl)propane]dihydrochloride (VA-044) (Aldrich, 98%), 1,3,5-trioxane (Aldrich, ≥99%) and *N,N*-dimethylformamide (DMF, Normapur, VWR, France) were used as received. *N,N*-Dimethylacrylamide (DMAm) (Aldrich, ≥99%) was distilled under reduced pressure before use. Deionized water was used for all aqueous polymerizations.

### 2.2. Synthesis of the PDMAm MacroRAFT Agents

Similar to a previously reported protocol [18], in a typical experiment (M3 in Table S1), 303 mg (1.14 mmol) RAFT agent (CTA-1), and 10.2 mg (0.062 mmol) of AIBN were dissolved in 22.9 mL DMF (Scheme S1). For the determination of the monomer consumption by ^1^H- NMR, 19 mg (0.21 mmol) of trioxane were added as an internal reference. After purging the solution with argon for 30 min in an ice bath, 5.40 mL (52.4 mmol) of degassed *N*,*N*-dimethylacrylamide (DMAm) were injected into the flask through an air-tight syringe. Afterwards, the flask was placed in a thermostated oil bath at 70 °C. To determine the monomer conversion, aliquots were periodically taken from the reaction medium and analyzed by ^1^H-NMR. The polymerization was quenched by exposure to air and cooling in an ice bath. The polymer was purified by precipitation in cold diethyl ether, and dried under reduced pressure at 50 °C. The SEC chromatograms in DMF (+LiBr 1 g L^−1^) are given in Figure S2.

### 2.3. Synthesis of PDMAm-b-PCMAm Diblock Copolymers in Water

In a typical experiment (Entry 7, Table 1), 53 mg (0.014 mmol) of PDMAm_36_-TTC, 500 µL of an initiator solution (15.7 mg of VA-044 (2,2′-azobis[2-(2-imidazolin-2-yl) propane]dihydrochloride) diluted in 5.03 g of water) (0.005 mmol) and 599 mg (5.45 mmol) of CMAm were dissolved in 6.20 g water in a 10 mL septum-sealed round bottom flask. The mixture was purged with argon for 30 min in a cold-water bath. The flask was immersed in a thermostated oil bath at 45 °C for 8 h. Monomer conversion was kinetically followed by taking aliquots from the reaction medium and analyzing them by ^1^H NMR. The polymerization was quenched by exposure to air and placing the flask into an ice bath.

### 2.4. Characterization Techniques

#### 2.4.1. Nuclear Magnetic Resonance Spectroscopy (NMR) 

^1^H-NMR spectra were recorded in DMSO-d_6_ (unless stated differently) at 300 K on a Bruker 300 MHz spectrometer in 5 mm diameter tubes.

#### 2.4.2. Size Exclusion Chromatography (SEC) 

The SEC measurements were carried out at 60 °C in DMF (+LiBr, 1 g L^−1^) as mobile phase at a flow rate of 0.8 mL min^−1^ and with toluene as a flow rate marker. All polymers were prepared at a concentration ranging from 5 to 10 mg mL^−1^, filtered through a 0.20 μm PTFE membrane; 100 μL of each solution were injected for each measurement for analysis. The separation system was composed of two PSS GRAM 1000 Å columns (8 × 300 mm; separation limits: 1 to 1000 kg mol^−1^) and one PSS GRAM 30 Å (8 × 300 mm; separation limits: 0.1 to 10 kg mol^−1^) coupled with a modular differential refractive index (RI) detector (Viscotek TDA, Malvern, France). Molar masses (*M*_n_, the number-average molar mass, *M*_w_, the weight-average molar mass) and dispersities (*Ð* = *M*_w_/*M*_n_) were calculated using the OmniSEC 5.12 software with a calibration curve based on narrow PMMA standards (from Polymer Standard Services, Mainz, Germany).

#### 2.4.3. Turbidimetry 

The turbidimetry measurements of copolymers in water were performed on a Cary 100 UV-Vis spectrophotometer (Agilent) equipped with a Peltier-type temperature control system by measuring the transmittance at a wavelength of 670 nm. The heating/cooling rate was maintained constant at 1 °C min^−1^. Samples were prepared at a concentration of 1 wt% by diluting the polymer dispersion in ultra-pure water. The cloud point temperature (T_CP_) was determined at the inflection point.

#### 2.4.4. Dynamic Light Scattering (DLS)

The DLS measurements were carried out on a Zetasizer Nano S90 from Malvern (France; 90° angle, 5 mW He–Ne laser at 633 nm) to determine the z-average particle diameter (*D*_z_) of diluted dispersions in water at 0.1 wt% (unless stated differently). Polydispersity indices (PDI) were determined using the cumulant method.

#### 2.4.5. Cryogenic Transmission Electron Microscopy (Cryo-TEM)

Polymer solutions were prepared at 1 wt% in ultra-pure water. Typically, 3 µL of the solution was deposited on a quantifoil grid. After removing the excess of solution with a Whatman filter paper, the grid was immediately frozen in liquid ethane. The observations were carried out at −180 °C with a JEM-2100 LaB_6_ microscope (JEOL) operating at 200 kV. The images were taken on a Gatan US 1000, 2k by 2k CCD camera.

#### 2.4.6. Small Angle X-ray Scattering Analyses (SAXS)

Selected samples were analyzed by small angle X-ray scattering (SAXS) measurements on the SWING beamline of the SOLEIL Synchrotron (Saint Aubin, France). The measurements were performed during two series of measurements at an energy of 7 keV (*λ* = 1.77 Å) (respectively at 12 keV (*λ* = 1.03 Å)), with an exposure time of 1000 ms and a gap time of 500 ms and measured by a two-dimensional CCD detector localized at a distance of 2131 mm (respectively 3500 mm) from the sample. The measurements were performed at different concentrations and different temperatures thanks to a thermostated capillary-holder device. Standard correction procedures were applied for X-ray beam transmission, signal subtraction of the 1.5 mm capillary filled with the solvent and detector efficiency. The softwares Foxtrot^®^ and SASview^®^ were used to achieve such data reduction. The data was fitted with the SASview^®^ software (http://www.sasview.org/, accessed in 15 May 2021). According to the morphologies observed by cryo-TEM the data were fitted with the form factor of a lognormal distribution of either spheres, cylinders or vesicles. The values obtained are summarized in Table 2 and the fits are displayed in SI, Figure S4.

## 3. Results and Discussion

### 3.1. Synthesis of PDMAm-b-PCMAm Diblock Copolymer Assemblies by RAFT-Mediated Aqueous Dispersion Polymerization

**Scheme 1:**
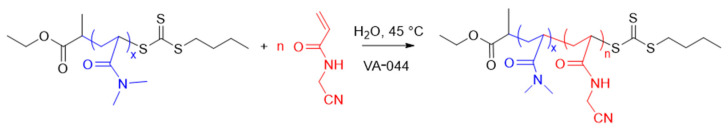
Synthesis route for the preparation of PDMAm-*b*-PCMAm diblock copolymers in water.

In order to produce PDMAm-*b*-PCMAm diblock copolymers self-assemblies in water, our synthetic approach relies on a PISA strategy, where CMAm is polymerized in water in the presence of hydrophilic PDMAm-TTC macroRAFT agents (Scheme 1) in conditions where self-assembly occurs during polymerization. In our previous study [11], UCST-type cloud points (T_CP_) between 54 °C and over 90 °C were determined (at 0.5 wt%) for PCMAm homopolymers depending on the number-average degree of polymerization (*DP*_n_ between 20 and 180). Recent experiments revealed that the UCST behavior, formerly mostly studied at 0.5 wt%, was also preserved at 10 wt%, which is actually close to the concentration at which PISA can be reached; for instance, for a PCMAm homopolymer with *DP*_n_ = 113, we determined visually a T_cp_ above 60 °C.

Therefore, the polymerization temperature was set to 45 °C, which is *a priori* significantly below the T_CP_ determined for the PCMAm homopolymers, while the CMAm monomer is soluble. These polymerization conditions should therefore lead to the in situ formation of nano-assemblies composed of diblock copolymers, as expected for a typical PISA process proceeding through an aqueous dispersion polymerization mechanism. 2,2′-Azobis[2-(2-imidazolin-2-yl)propane]dihydrochloride (VA-044) was chosen as water-soluble radical initiator for its low decomposition temperature (10 h half-life dissociation temperature in water = 44 ℃) [20].

In three series of experiments, we systematically varied the length of the PDMAm-TTC macroRAFT agents and the PCMAm block. Therefore, three PDMAm-TTC macroRAFT agents with *DP*_n_ = 13, 23 and 36, respectively, were synthesized according to a reported procedure [18] and well-defined polymers with molar mass dispersities *Ð* below 1.2 were obtained (see Table S1 and Figure S2). For the synthesis of the PDMAm-*b*-PCMAm diblock copolymers, the length of the PCMAm block was tuned by the initial monomer to PDMAm-TTC molar ratio. The polymerization conditions and results are summarized in Table 1. Generally, in all experiments high monomer conversions were reached. 

**Figure 1:**
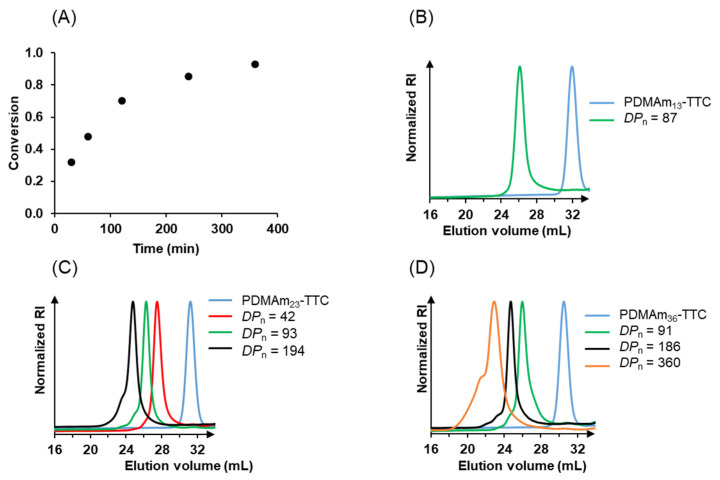
(**A**) Monomer conversion monitored by ^1^H NMR in DMSO-d_6_ for PDMAm_23_-*b*-PCMAm_93_ (*Entry 3*, Table 1); Normalized size exclusion chromatograms of PDMAm macroRAFT agents and the resulting diblock copolymers using PDMAm macroRAFT agents of various *DP*_n_: (**B**) *DP*_n_ = 13; (**C**) *DP*_n_ = 23; (**D**) *DP*_n_ = 36. The *DP*_n_ reported in the figures is the *DP*_n_ of the PCMAm block determined via the monomer conversion.

Size exclusion chromatography (SEC) indicated the quantitative extension of PDMAm-TTC to PDMAm-*b*-PCMAm diblock copolymers with a complete shift of the PDMAm macroRAFT agent signal (Figure 1). Dispersities were excellent (*Ð* ≤ 1.2), except for *sample 4* (PDMAm_23_-*b*-PCMAm_194_) and in particular *sample 7* (PDMAm_36_-*b*-PCMAm_360_), for which a shoulder at the higher molar mass side - generally attributed to the formation of dead chains by chain recombination - was observed.

### 3.2. Impact of DP_n_ of PDMAm and PCMAm on Colloidal Stability and Morphology

Macroscopically, important differences were observed for the three series of experiments, prepared with the three different macroRAFT agents (Table 1). Using the shortest macroRAFT agent (*DP*_n_ = 13), the reaction medium phase separated during polymerization even though a relatively short PCMAm block was targeted (*DP*_0_ = [CMAm]_0_/[TTC]_0_ = 100). In line with the literature [21,22], this observation should be explained by an insufficient stabilization due to a too short stabilizer. Indeed, a stable dispersion was obtained when the experiment was repeated with the two longer macroRAFT agents, with *DP*_n_ = 23 and 36, *samples 3 and 5*. When the length of the solvophobic PCMAm block was increased to *DP*_0_ ~200, we observed again a macroscopic phase separation for *sample 4* prepared with the macroRAFT agent of *DP*_n_ = 23. Again, increasing the length of the stabilizer led to a stable dispersion, *sample 6*. It can thus be concluded that the longest PDMAm stabilizer with *DP*_n_ = 36 is the most suitable one to polymerize CMAm in water leading to the formation of stable dispersions over a large molar mass range of the PCMAm block.

**Figure 2:**
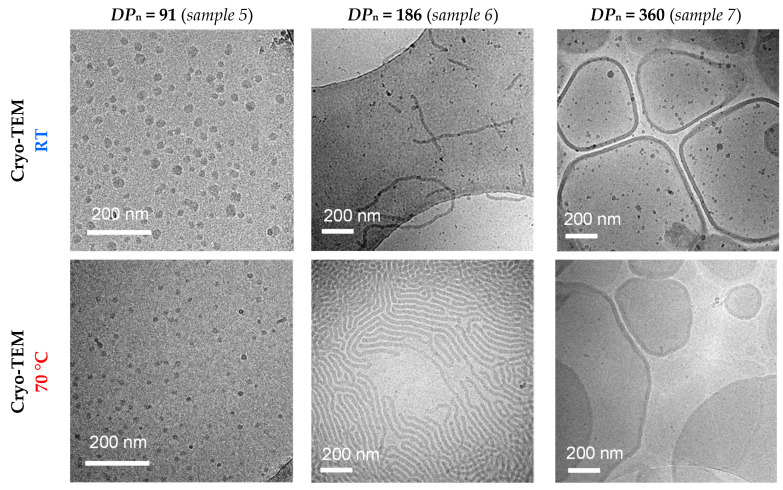
Representative cryo-TEM images of PDMAm_36_-*b*-PCMAm*_x_* dispersions, prepared at room temperature or at 70 °C. Sample concentration = 1 wt% in water (except for *DP*_n_ = 186, prepared at RT at <0.1 wt% and at 70 °C at 0.1 wt%).

In order to determine the size and morphology of the colloids, the dispersions were analyzed by cryo-TEM as shown in Figure 2 (top, deposition at room temperature). For *sample 5* with the shortest PCMAm block, tiny spheres with an average diameter of 14 nm were observed. In classical PISA, higher order morphologies can be obtained by progressively increasing the solvophobic block length-provided that chain reorganization is possible. Indeed, for the intermediate PCMAm block length (*DP*_n_ = 186), nanoworms with an average diameter of 21 nm were observed. Furthermore, this sample exhibited an increased viscosity, which is commonly observed for worm dispersions [23,24,25]. For the longest PCMAm block (*DP*_n_ = 360), large vesicles—with a membrane thickness of 22 nm—were clearly observed. The diameter of the nanospheres and worms, and the membrane thickness of the vesicles increased thus with increasing *DP*_n_ of PCMAm, as expected.

**Figure 3:**
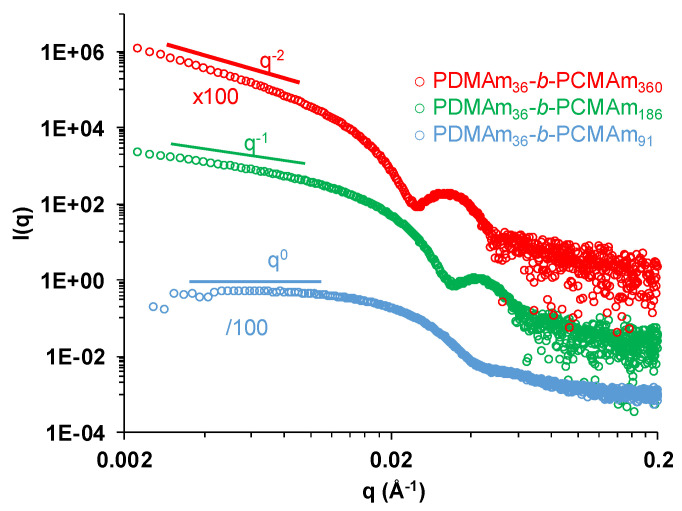
SAXS intensity (I(q)) versus scattering vector (q) for PDMAm_36_-*b*-PCMAm_x_ (*samples 5, 6 and 7*) in water at 1 wt% at 25 °C.

In order to confirm these results, SAXS and DLS measurements were also performed at 25 °C (Figure 3 and Figure S3, respectively) and the results are summarized in Table 2. The SAXS measurements performed at 1 wt% in water confirmed the sphere, worm and vesicle shapes (see fits Figure S4). Their modelled diameters and the thickness of the vesicle membrane were in good agreement with those determined by cryo-TEM (Table 2). 

The DLS measurements (Table 2) further confirmed the diameter of the vesicles. Concerning the spherical nano-objects, the size determined by DLS was significantly higher than the one determined by cryo-TEM. This difference might in part be explained by the absence of contrast of hydrated chains in cryo-TEM. Therefore, we may assume that the diameters determined by cryo-TEM comprises only the dehydrated core of the aggregate while DLS determined a hydrodynamic diameter of the whole object.

### 3.3. Thermoresponsive Behavior

We have previously demonstrated that PCMAm chains possess a UCST-type solubility behavior in water. We therefore investigated whether the PDMAm-*b*-PCMAm diblock copolymer assemblies obtained directly in water showed any temperature-induced transitions.

**Figure 4:**
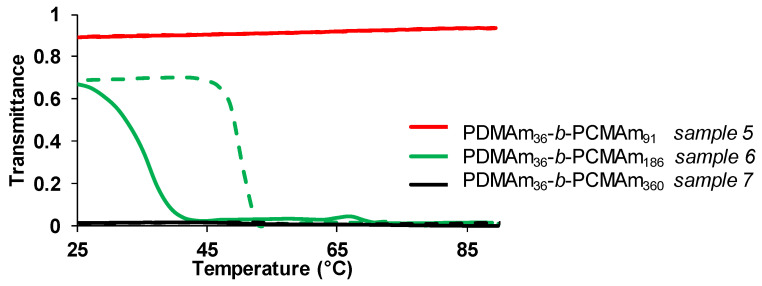
Turbidity curves of PDMAm-*b*-PCMAm dispersions prepared at 1 wt% in water (λ = 670 nm, 1 °C min^−1^) (Table 1). First cooling: solid lines; second heating: dashed lines.

First, the series prepared with the longest stabilizer (PDMAm_36_-TTC, *Samples 5, 6* and *7*) was analyzed by temperature-dependent turbidimetry measurements at 1 wt% in water. Figure 4 does not show any significant temperature dependency for *Samples 5* and *7* (spheres and vesicles). In contrast, a clear decrease in transmittance upon heating was observed for the worm sample (*Sample 6*). The temperature-induced change in transmittance was reversible but presented a large hysteresis.

In order to better understand the influence of temperature on these samples, heated samples were also characterized by cryo-TEM, DLS and SAXS. For the cryo-TEM experiments, the samples were heated to 70 °C before rapid deposition on the cryo-TEM grids. The results are illustrated in Figure 2 (bottom). For *Samples 5* and *6* (spheres and worms), the comparison of samples deposited at 70 °C and room temperature Figure 2 (bottom and top) indicated that the heating did not induce any significant change in morphology nor in size (Table 2). The observed change in turbidity for the worm *Sample 6* should thus not be related to a change in morphology. It might rather be explained by a secondary aggregation of the initial worms, leading to an increase in turbidity through the formation of large scattering objects. Such secondary aggregation, which should be triggered by a diminished colloidal stability at higher temperature, might even end-up with the partial precipitation of the sample, as already observed in the literature for a similar system [18]. Actually, SAXS measurements performed on *Sample 6* (worms) at 1 wt% at 70 °C (Figure S5A) confirmed that the system becomes colloidally unstable upon heating since the diffractogram of the resulting sample was similar to that of pure water. Indeed, in contrast to turbidity measurements, SAXS analyses were actually performed without stirring. Thus, the absence of signal in scattering intensity must be attributed to polymer precipitation to the capillary bottom that occurs over time when the sample is not stirred. Interestingly, SAXS analyses performed at 70 °C at lower concentration (0.5 wt% and 0.1 wt%, Figure S5B,C) did not induce precipitation of the sample (since a scattering signal could be observed at 70 °C). Furthermore, these experiments showed that the process was fully reversible as the diffractogram recorded at 20 °C (after cooling back to 20 °C) perfectly overlayed the initial one.

**Figure 5:**
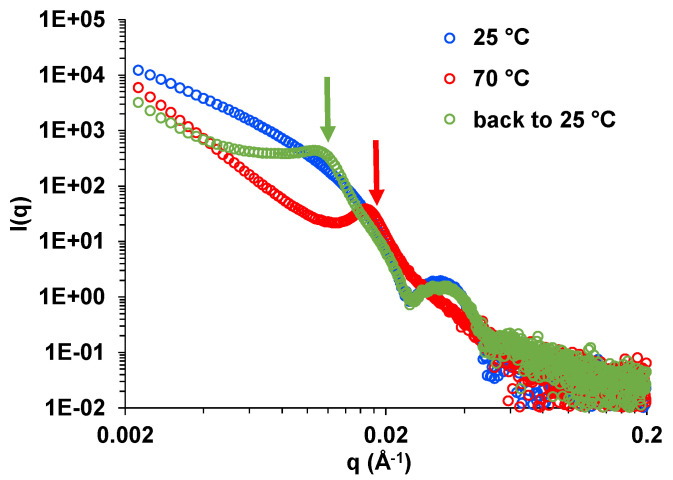
SAXS intensity (I(q)) versus scattering vector (q) for PDMAm_36_-*b*-PCMAm_360_ (*Sample 7*) in water at 1 wt%, at 25 °C (in blue), 70 °C (in red) and back to 25 °C (in green). The arrows designate *d*, the reciprocal length corresponding to the inter-vesicle distance.

Concerning *Sample 7* (vesicles), in agreement with the turbidity measurements, the DLS measurements of *Sample 7* (Figure S3C) indicated the presence of large aggregates at both 25 and 65 °C, with no significant temperature-dependency. Cryo-TEM analyses of the sample deposited at 70 °C (Figure 2, bottom) suggested that the heating process might however have changed the structure of the vesicles′ membrane. Whereas clearly defined at RT (Figure 2, top), on selected images the membrane seems altered and partially disaggregated at 70 °C (Figure S6). However, on most regions of the grid, pristine vesicles were observed (Figure 2, bottom), which makes it difficult to conclude whether heating has truly an impact on the vesicle membrane or not. SAXS analyses at 1 wt%, performed successively at 25 °C, 70 °C and cooled back at 25 °C (Figure 5), supported our assumption that the sample might evolve with temperature. Indeed, a clear change in the scattering profile was observed, when the pristine sample analyzed at 25 °C was heated to 70 °C. In contrast to *sample 6*, visually, no macroscopic precipitation was observed. The clear evolution of the diffractogram with temperature seemed rather to correspond to the appearance of a structure factor characteristic for interactions between vesicles. At this stage, we assume that this change in the scattering profile could be attributed to an increase of attractions between vesicles potentially leading to the formation of secondary aggregates when the sample is heated. Indeed, the peak observed at 70 °C on the scattering intensity profile corresponds to a characteristic distance between interacting. This peak allowed us to calculate a characteristic distance *d* of 35.0 nm (d=2π/q) between objects. Interestingly, after cooling the sample back to room temperature, the scattering profile became closer to the initial one, indicating that the temperature-induced changes are at least partially reversible on the timescale of the experiment. These vesicles seem still partially aggregated, but the distance between them increased as evidenced by the characteristic peak, displaying an inter-vesicle distance of 52.3 nm. Interestingly, SAXS measurements performed at lower concentration, namely 0.5 wt% and 0.1 wt% (Figure S7), showed that the aggregation process-still visible at 70 °C-was totally reversible at these lower concentrations.

These combined analyses let us conclude that-upon heating-the worms (*sample 6*) aggregate into larger, secondary aggregates that precipitate with time, while the vesicles (*Sample 7*) remained colloidally dispersed even at high temperature, at least at the time scale of the experiment.

Concerning the spheres (*Sample 5*), turbidity, cryo-TEM and DLS (Figure S8C) performed at 1 wt% suggested that they were not sensitive to modification in temperature. However, we cannot exclude an impact of concentration on the stability, reorganization or destabilization of the nano-objects with temperature. Actually, whereas the DLS analyses of the spheres (*Sample 5*) clearly did not reveal any temperature-induced transition (at least up to 65 °C) at 1 wt% in water (Figure S8C), DLS at 0.1 wt% (Figure S3A) indicated a typical UCST-type dissociation of the aggregates upon heating: at 65 °C the count rate was significantly decreased and no precipitation was observed. SAXS analyses at 0.1 wt% (Figure S9) confirmed this conclusion. The results highlight the crucial importance of the polymer concentration on the UCST transition as already observed for certain thermoresponsive polymers [18,26]. In contrast, for the worms and vesicles, decreasing the concentration from 1 to 0.1 wt% (DLS: Figure S3; SAXS: Figure S5B, S5C and S7) did not lead to the dissociation of the assemblies. The absence of a UCST-type behavior is certainly related to the longer PCMAm block, which should increase the transition temperature above 90 °C [11].

**Figure 6:**
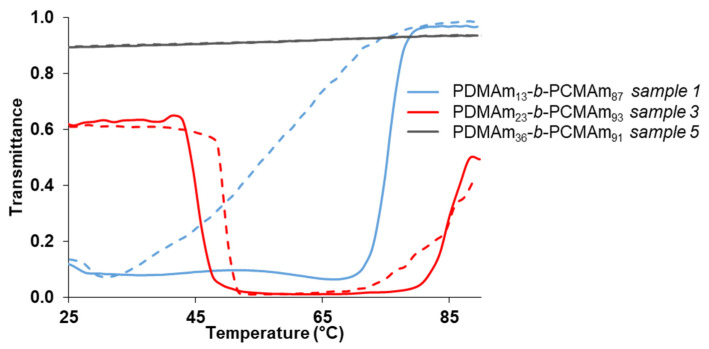
Turbidity curves of PDMAm*_x_*-*b*-PCMAm_~90_ dispersions (Table 1) prepared at 1 wt% in water (λ = 670 nm, 1 °C min^−1^). First cooling: solid lines; second heating: dashed lines.

In order to study the impact of the PDMAm-stabilizer length on the temperature-sensitivity, in a second series of experiments, we also screened the temperature-dependent turbidity for *Samples 1* and *3* (PDMAm_13_-*b*-PCMAm_87_ and PDMAm_23_-*b*-PCMAm_93_ respectively), which possess a similar, relatively short PCMAm block (*DP*_n_ ~90), but considerably shorter stabilizers than *Sample 5* (PDMAm_36_-*b*-PCMAm_91_). The results are summarized in Figure 6.

We should remind here, that *Sample 1* (PDMAm_13_-*b*-PCMAm_87_) was colloidally unstable and phase-separated during polymerization and during storage at 4 °C at 10 wt%. Turbidity measurements at 1 wt% (Figure 6) combined with DLS at 1 wt% (Figure S8A) revealed a UCST-type transition with a cloud point (T_CP_) about 76 °C (1^st^ cooling). The DLS measurements at temperatures 90 °C at 1 wt% showed a very low scattering intensity close to the one of water, supposing the complete dissolution of the polymer chains, as expected for a UCST-type polymer and observed for the PCMAm homopolymer. The determined T_CP_ is actually slightly lower that the T_CP_ determined for a PCMAm homopolymer of similar molar mass (*DP*_n_ = 73: T_CP_ (0.5 wt%) = 85 °C) [11]. This observation is in agreement with the literature, where it has been reported that the presence of a hydrophilic polymer block increases the overall solubility of the copolymer thus lowering the T_CP_ [18].

**Figure 7:**
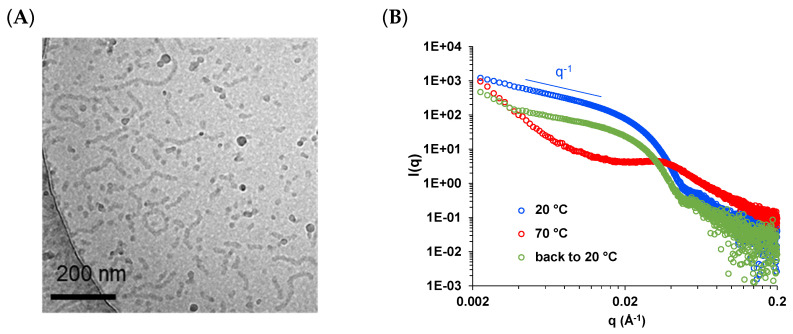
PDMAm_23_-*b*-PCMAm_93_ (*sample 3*) aqueous dispersions: (**A**) Representative cryo-TEM image prepared at room temperature at 1 wt%; (**B**) SAXS profile at 1 wt% at 20 °C (in blue), 70 °C (in red) and back to 20 °C (in green).

For *Sample 3* (PDMAm_23_-*b*-PCMAm_93_), the turbidimetry measurements-performed under stirring at 1 wt%-showed a more complex temperature transition, with a decrease in transmittance above 40 °C followed by an increase in transmittance above 80 °C, which was not caused by a macroscopic precipitation of the sample. At room temperature, macroscopically, the sample was colloidally stable and cryo-TEM analysis indicated that it was composed of a mixture of worms of different lengths-the shortest one resembling spheres-with an average diameter of *D*_n_ = 14.7 nm (Figure 7A). The morphology was also studied by means of SAXS as illustrated on Figure 7B. The experiments at 20 °C (at 1 wt%), confirmed the worm-like morphology observed by cryo-TEM. Upon heating to 70 °C the scattering profile evolved towards aggregated fibers*,* with the appearance of structure factor displaying a broad characteristic peak, corresponding to a distance of around 21 nm (*d* = 2*π*/*q*) between aggregated fibers. Interestingly, when the sample was cooled back to room temperature, this aggregation seems to be partially reversible, at the timescale of the experiment. Indeed, a worm-like form factor (q^−1^) similar to the one obtained originally was measured, however with a reduced overall intensity, indicating less scattering light objects compared to the original sample.

DLS analyses at 1 wt% were not reliable upon heating to 70 °C as the sample phase separated during the measurement, while at 0.1 wt% no phase separation was observed (Figure S10). Instead, Figure S10A showed a reversible increase in diameter when the sample was heated from 20 to 70 °C, which is in agreement with the SAXS analyses. In more details, at 0.1 wt%, we observed the presence of small sub-100 nm aggregates at 20 °C, which evolved towards larger aggregates upon heating to 70 °C. When the sample was further heated to 90 °C the scattering intensity dropped to a value close to water. This decrease might be explained by either (a) the molecular dissolution of the assemblies or (b) a macroscopic precipitation of the polymer toward the bottom of the unstirred cuvette (which was not observed but might be difficult to observe by the naked eye in view of the low sample concentration). At higher concentration, at 1 wt%, heating the sample to 70 °C caused a destabilization of the system and the precipitation of the polymer visible at the bottom of the DLS cuvette. The remaining polymer formed micrometer-sized aggregates that precipitated over time. Rapid heating of the sample from RT to 90 °C led also to a very low scattering intensity and small objects below 10 nm were detected. Again, this could be attributed to either the molecular dissolution or macroscopic precipitation of the sample. Visually, we did not detect any deposit, and we can thus conclude that the polymer molecularly dissolved at 90 °C. Finally, at both sample concentrations (0.1 and 1 wt%), the initial size of the small aggregates was recovered when the samples were cooled back to 20 °C; the transition is thus reversible (Figure S10) as already indicated by turbidimetry. In view of theses combined analyses, the apparent LCST-type transition, might therefore be explained by the destabilization of the colloidal system [27]. At both concentrations, upon heating from 20 to 70 °C, the initial aggregates form larger aggregates through a secondary aggregation mechanisms, which are nanometric and remain colloidally stable at 0.1 wt%, but become micrometric and colloidally unstable at 1 wt%. When the samples are further heated, the apparent UCST transition observed by turbidimetry above ~90 °C should be attributed to the dissociation and solubilization of the polymer chains.

## 4. Discussion and Conclusions

In summary, we demonstrated the possibility to synthesize in water-via RAFT-mediated PISA-block copolymer nanoassemblies with PCMAm as a hydrophobic block, using hydrophilic PDMAm-TTC macroRAFT agents. Overall, we demonstrated the formation of well-defined PDMAm-*b*-PCMAm diblock copolymers with good polymerization control. We have shown that a sufficient stabilizer/hydrophobic block ratio was crucial for the preparation of colloidally stable dispersions.

**Figure 8:**
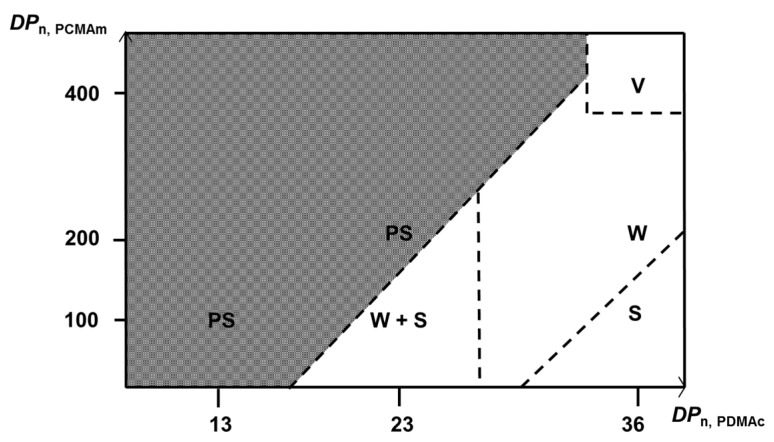
Supposed phase diagram for PDMAm-*b*-PCMAm diblock copolymers prepared at 10 wt% monomer concentration in water. PS = phase separation, S = spheres, W = worms, V = vesicles.

In Figure 8, we attempted to rationalize the influence of the respective block length, i.e., *DP*_n_, of the hydrophilic stabilizing block, PDMAm, and the PCMAm hydrophobic block, on the colloidal stability and morphologies obtained during the PISA process.

As indicated by the gray zone in the pseudo-phase diagram, short PDMAm blocks lead to unstable dispersions at the end of the polymerization, whereas large PDMAm block (*DP*_n_ = 36) enabled the synthesis of stable nano-objects, of which the morphology could be tuned from spheres to worms to vesicles by adjusting the *DP*_n, PCMAm_ (Figure 2).

Even though short PCMAm blocks were shown to possess an UCST when studied as a homopolymer [11], most of the diblock copolymers studied here did not exhibit any typical UCST behavior. Only diblocks with a short PCMAm block (*DP*_n,PCMAm_ < 100) dissociated at low concentration upon heating. Yet for higher *DP*_n,PCMAm_s, the diblock copolymer assemblies did not dissociate upon heating, nor displayed any morphological transition. At sufficiently high temperature, they rather exhibited a temperature-dependent secondary aggregation of primary particles, which explained the observed decrease in transmittance in the turbidimetry measurements.

We believe that our development of an aqueous synthesis pathway towards PCMAm-based copolymers will pave the way towards many other PCMAm-based responsive nano-objects, notably thermoresponsive nanogels that will be highly valuable for biomedical applications.

## Figures and Tables

**Table 1:** Experimental conditions and results for the aqueous polymerizations of CMAm in the presence of various PDMAm-TTC macroRAFT agents #.

*Sample*	[CMAm]_0_/ [TTC]_0_/[A]_0_^a^	Time (h)	Conv. ^b^ (%)	*DP*_n,th_^c^	*M*_n,th_^c^ (kg mol^−1^)	SEC DMF		Visual Aspect ≤45 °C ^e^
						*M*_n_^d^ (kg mol^−1^)	*Đ* ^d^	
***PDMAm-macroRAFT DP_n_ = 13***								
1	100/1/0.23	2.8	87	87	11.1	22.1	1.15	Turbid, phase separation during polymerization
***PDMAm-macroRAFT DP_n_ = 23***								
2	50/1/0.24	2.8	83	42	7.2	12.8	1.14	Transparent
3	100/1/0.15	6	93	93	12.8	24.4	1.15	Slightly turbid
4	225/1/0.32	4	86	194	20.8	34.9	1.54	Phase separation during polymerization
***PDMAm-macroRAFT DP_n_ = 36***								
5	100/1/0.45	8.3	91	91	13.8	23.4	1.16	Slightly turbid
6	200/1/0.35	5	93	186	24.3	42.4	1.23	Slightly turbid
7	400/1/0.35	8	90	360	43.4	83.5	1.63	Milky solution

# Polymerizations performed in water at 45 °C in presence of PDMAm_x_-TTC using VA-044 as a radical initiator at targeted solids contents of 10 wt%. ^a^ Initial molar ratio of CMAm/ PDMAm-TTC/ VA-044. ^b^ Determined by ^1^H NMR. ^c^ Theoretical number-average degree of polymerization, *DP*_n,th_, and theoretical number-average molar mass, *M*_n,th_, determined via the monomer conversion. ^d^ Number-average molar mass *M*_n_, and dispersity, *Ð*, determined by SEC in DMF (+LiBr 1 g L^−1^) with a PMMA calibration. ^e^ Visual observations were done at 45 °C and 4 °C; no difference was noticed at both temperatures.

**Table 2:** Dimensions of the cylindrical micelles deduced from DLS measurements, cryo-TEM analyses and the fit of SAXS data.

*Sample* *Name*	*DP*_n_ PCMAm	Morphology	*D*_z_^a^ DLS @25 °C (PDI) ^b^	*D*_n_^c^ Cryo-TEM @RT (σ) ^d^	*D*_n_^c^ Cryo-TEM @70 °C (σ) ^d^	*D* ^e^ SAXS @25 °C (σ’) ^f^	Vesicle Membrane Thickness Cryo-TEM @RT (σ) ^d^	Vesicle Membrane Thickness SAXS @ 25 °C (σ’) ^f^
5	91	Sphere	29 nm (0.23)	14 nm (1.9 nm)	15 nm (1.2 nm)	18 nm (0.15)	-	-
6	186	Worm	153 nm * (0.23)	21 nm (2.4 nm)	19 nm (1.5 nm)	22 nm (0.10) [>300 nm] ^g^	-	-
7	360	Vesicle	551 nm (0.16)	592 nm (192 nm)	391 nm (110 nm)	>300 nm ^g^	22 nm (2.5 nm)	26 nm (0.1)

^a^ DLS realized at 0.1 wt% in water (see Figure S3). ^b^ Polydispersity index determined with a cumulant method. ^c^ *D*_n_ = number-average diameter determined on 20 representative nano-objects by cryo-TEM at 1 wt% in water. ^d^ Standard deviation. ^e^ *D* = median diameter determined by SAXS measurements realized at 1 wt% in water, modelled by form factors of objects displaying lognormal distributions of dimensions (see Figure S4). ^f^ Polydispersity determined with a lognormal distribution. ^g^ Length of the worms or vesicle size cannot be determined by SAXS since it exceeds the analysis window. * Sphere-equivalent diameter.

## Data Availability

Data sharing not applicable.

## Supplementary Materials

The following data are available online at https://www.mdpi.com/article/10.3390/polym13244424/s1. Scheme S1: Synthesis route for the PDMAm-TTC macroRAFT agents via RAFT-mediated solution polymerization in the presence of CTA-1; Table S1: Experimental conditions and results for the RAFT-mediated polymerization of DMAm in DMF at 70 °C; Figure S1: ^1^H-NMR spectrum of M3 (Table S1) in CDCl_3_; Figure S2: SEC chromatograms in DMF (+LiBr) of the macroRAFT agents with different *DP*_n_s; Figure S3: Temperature-dependent size distribution determined by DLS at 0.1 wt% in water for *Samples 5, 6* and *7* (PDMAm_36_-*b*-PCMAm_x_); Figure S4: SAXS intensity (I) versus scattering vector (q) for selected diblock PDMAm-*b*-PCMAm samples at 1 wt% in water at 25 °C (*Samples 5, 6* and *7*, Table 1); Figure S5: SAXS intensity (I) versus scattering vector (q) for PDMAm_36_-*b*-PCMAm_186_ (*Sample 6*) in water at (A) 1 wt%; (B) 0.5 wt% and (C) 0.1 wt% in water at 25 °C (or 20°C) and 70 °C; Figure S6: Selected cryo-TEM image of *Sample 7* (PDMAm_36_-*b*-PCMAm_360_) prepared at 70 °C, showing the presence of other morphologies than vesicles. Sample concentration = 1 wt% in water. Figure S7: SAXS intensity (I) versus scattering vector (q) for PDMAm_36_-*b*-PCMAm_360_ (*Sample 7*) in water at (A) 0.5 wt% and (B) 0.1 wt% at 25 °C (dark blue), 70 °C (red) and back to 25 °C (light blue). Thearrows designate d, the reciprocal length corresponding to the inter-vesicle distance; Figure S8: Size distributions determined by DLS of (A) PDMAm*_13_*-*b*-PCMAm_~90_ (*Sample 1*), (B) PDMAm_23_-*b*-PCMAm_90_ (*Sample 3*) and (C) PDMAm_36_-*b*-PCMAm_91_ (*Sample 5*) dispersions prepared at 1 wt% in water at various temperatures; Figure S9: SAXS intensity (I) versus scattering vector (q) for PDMAm_36_-*b*-PCMAm_91_ (*Sample 5*) in water at 0.1 wt% at 25 °C (dark blue), 70 °C (red) and back to 25 °C (light blue); Figure S10: Size distribution of *Sample 3* (PDMAm_23_-*b*-PCMAm_93_) at (A) 0.1 wt% and (B) 1 wt% in water determined by DLS at different temperatures (solid lines, first heating, dashed lines first cooling). At 90 °C a very low scattering intensity was detected (kcps = 6 and 50, respectively for 0.1 wt% and 1 wt%).
