# Supplementary material for: Synthesis and Characterization of Temperature-Responsive N-Cyanomethylacrylamide-Containing Diblock Copolymer Assemblies in Water
Source: Polymers (Basel). 2021 Dec 16;13(24):4424. doi: 10.3390/polym13244424 (PMC8707179; doi:10.3390/polym13244424)
Supplement: Supplementary file 1 [file polymers-13-04424-s001.zip › polymers-1504540-supplementary.pdf]

Supplementary Information

Synthesis and characterization of temperature-responsive *N*-cyanomethylacrylamide-containing diblock copolymer assemblies in water

Nicolas Audureau[1], Fanny Coumes[1], Clémence Veith[1], Clément Guibert[2], Jean-Michel Guigner[3], François Stoffelbach[1]*, Jutta Rieger[1]*

[1] Sorbonne Université & CNRS, UMR 8232, Institut Parisien de Chimie Moléculaire (IPCM), Polymer Chemistry Team, 4 Place Jussieu, 75252 Paris Cedex 05, France

jutta.rieger@sorbonne-universite.fr; francois.stoffelbach@sorbonne-universite.fr

[2] Sorbonne Université, CNRS, Laboratoire de Réactivité de Surface (LRS), 4 Place Jussieu, 75252 Paris Cedex 05, France

[3] Sorbonne Université & CNRS, UMR 7590, Institut de Minéralogie, de Physique des Matériaux et de Cosmochimie (IMPMC)-IRD-MNHN, 75252 Paris Cedex 05, France

1. Synthesis of the PDMAm macroRAFT agents

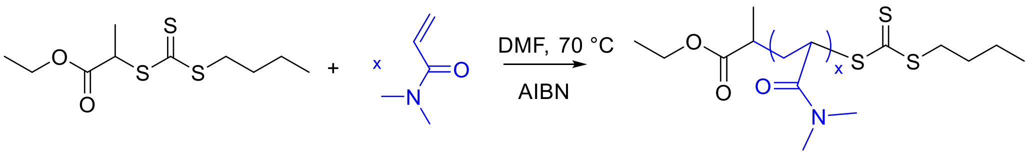

Scheme S1. Synthesis route for the PDMAm-TTC macroRAFT agents *via* RAFT-mediated solution polymerization in the presence of CTA-1.

Table S1. Experimental conditions and results for the RAFT-mediated polymerization of DMAm in DMF at 70 °C#

**Entry**	$\frac{[\text{DMAm}]_0}{[\text{CTA-1}]_0}$ [a]	**[DMAm]$_0$** (mol L_{DMF}^{-1})	**Time** (min)	**Conv.**[b] (%)	$DP_{n,th}$[c]	DP_n[b]	$M_{n,th}$[d] (kg mol^{-1})	**SEC DMF**[e] M_n[e] (kg mol^{-1})	$Đ$[e]
M1	23	2.5	80	59	14	13	1.55	1.35	1.12
M2	29	2.4	93	64	19	23	2.55	2.22	1.09
M3	46	2.3	210	74	34	36	3.83	3.19	1.09

#Polymerizations were performed in the presence of a RAFT agent (CTA-1) using AIBN as a radical initiator at an initial molar ratio of CTA-1/AIBN: 1/0.05. [a] Initial monomer/CTA-1 molar ratio. [b] Determined by ^{1}H NMR (for the determination of the DP_n of M3 see Figure S1). [c] Theoretical number-average degree of polymerization, $DP_{n,th}$ calculated using the experimental conversion. [d] Theoretical number-average molar mass, $M_{n,th}$, calculated using $DP_{n,th}$. [e] Number-average molar mass, M_n and dispersity, $Đ$, determined by SEC in DMF (+ LiBr 1g L^{-1}) with a PMMA calibration.

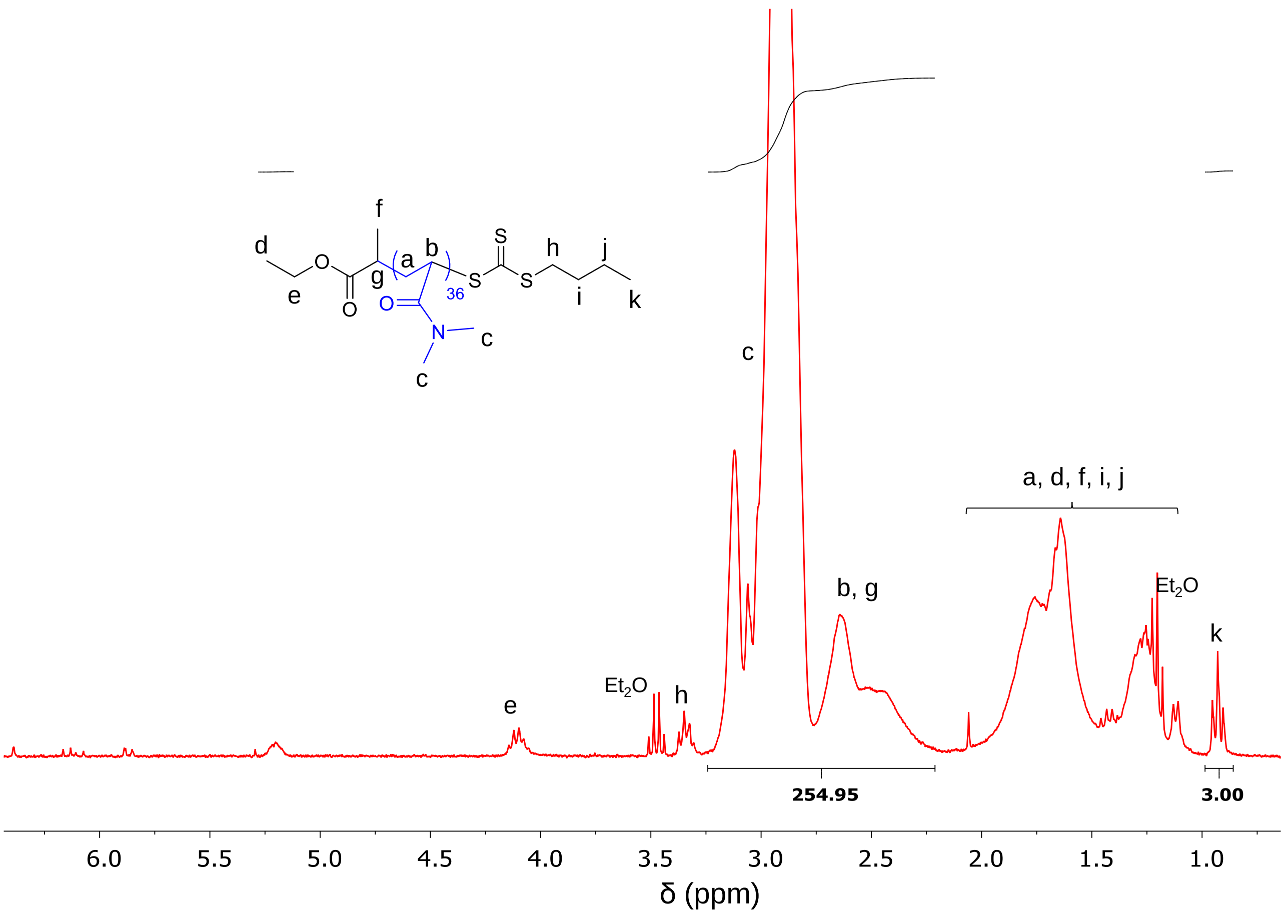

Figure S1. ^{1}H NMR spectrum of M3 (Table S1) in $CDCl_3$.

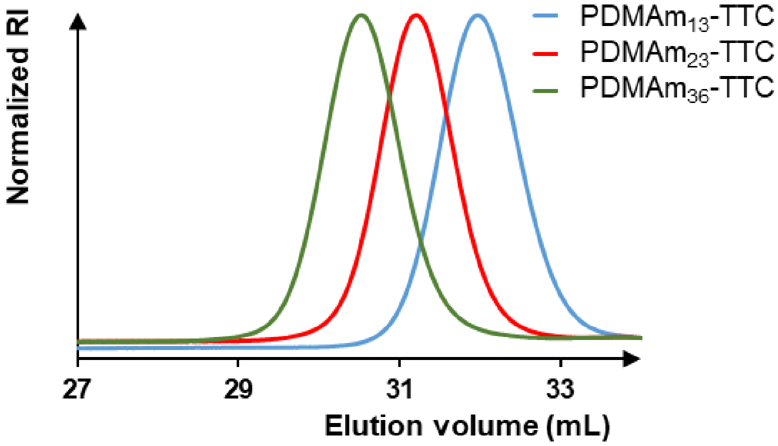

Figure S2. SEC chromatograms in DMF (+ LiBr) of the macroRAFT agents with different DP_ns.

2. *Supplementary data*

(A) DP_n = 91 (*sample 5*) (B) DP_n = 186 (*sample 6*) (C) DP_n = 360 (*sample 7*)

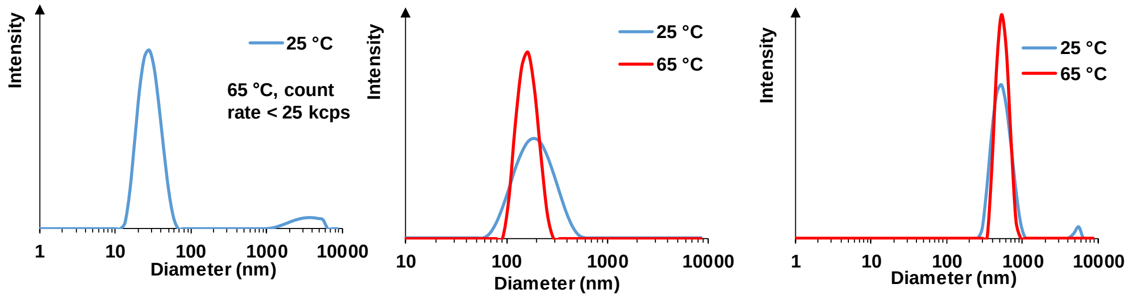

Figure S3. Temperature-dependent size distribution determined by DLS at 0.1 wt% in water for *samples 5, 6 and 7* (PDMAm$_{36}$-*b*-PCMAm$_x$).

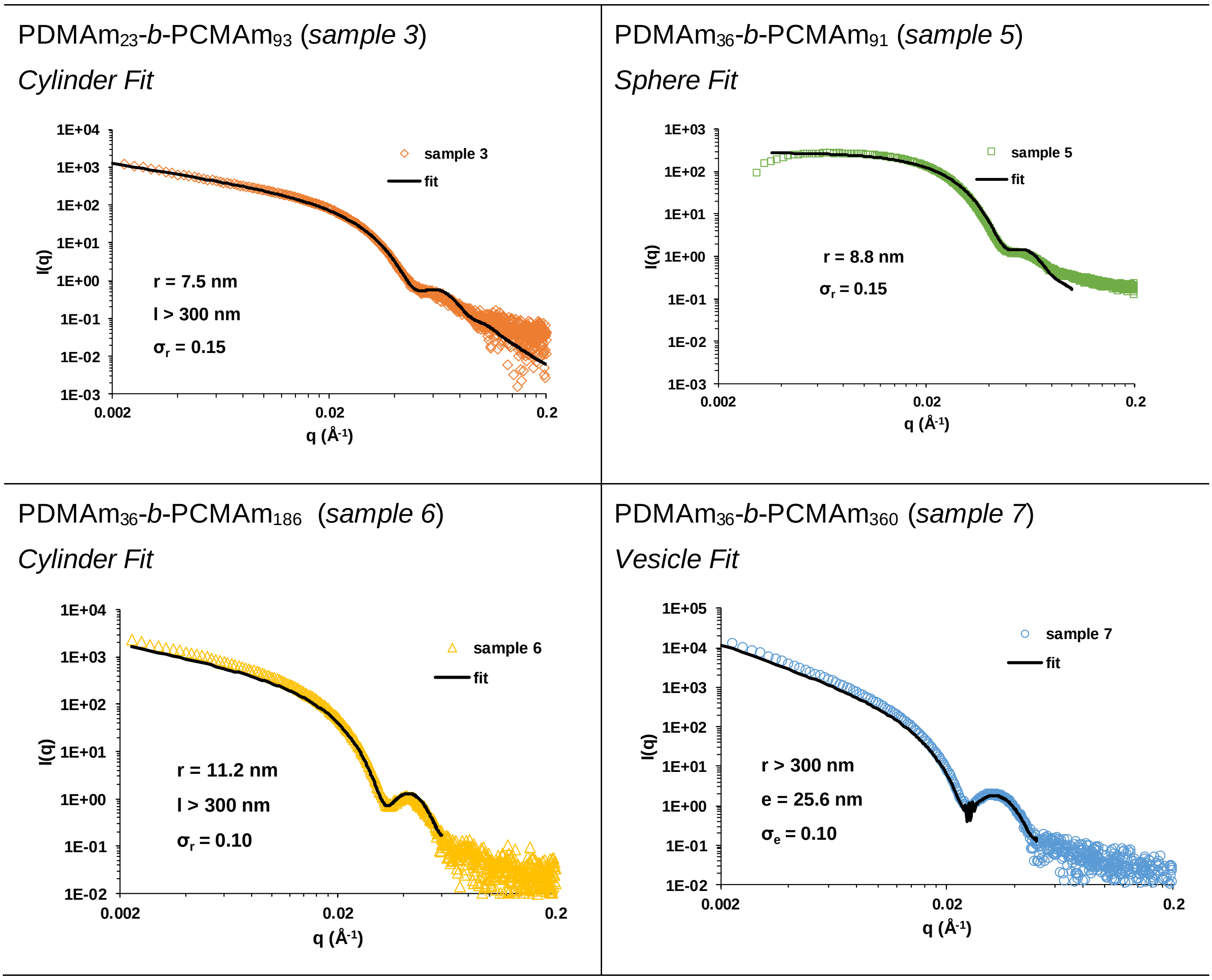

Figure S4. SAXS intensity (I) versus scattering vector (q) for selected diblock PDMAm-*b*-PCMAm samples at 1 wt% in water at 25 °C (*samples 3, 5, 6 and 7*, Table 1).

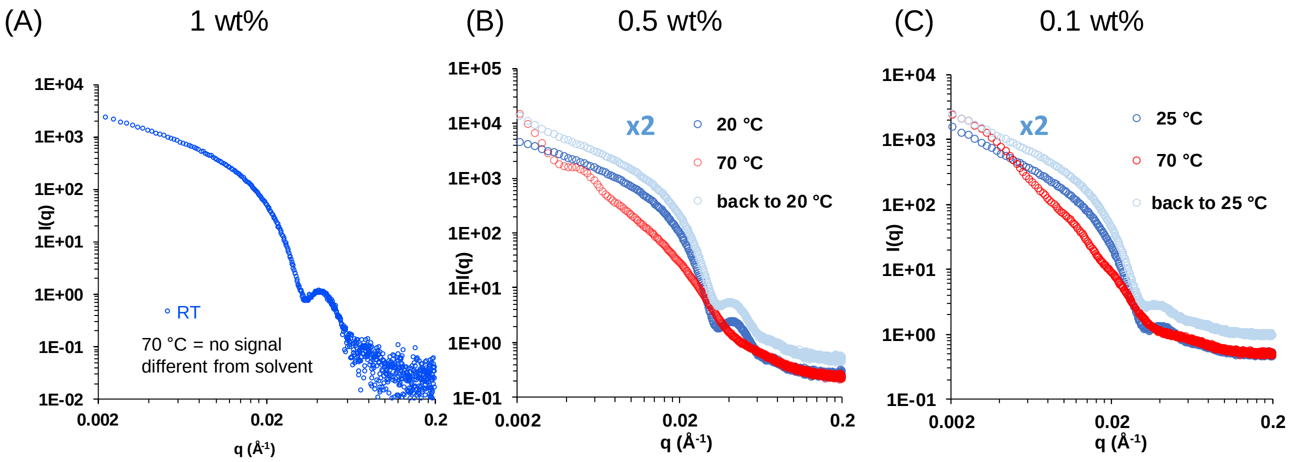

Figure S5. SAXS intensity (I) versus scattering vector (q) for PDMAm36-*b*-PCMAm186 (*sample 6*) in water at (A) 1 wt%; (B) 0.5 wt% and (C) 0.1 wt% in water at 25 °C (or 20°C) and 70 °C. For the samples cooled back after heating, the intensity was multiplied by a factor of 2 in order to visualize the signal, since it is a perfect overlay with the initial sample.

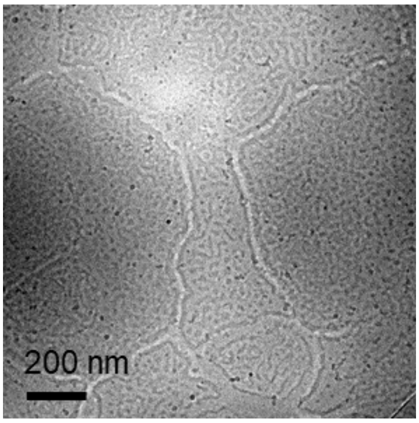

Figure S6. Selected cryo-TEM image of *sample 7* (PDMAm36-*b*-PCMAm360) prepared at 70 °C, showing the presence of additional morphologies different from standard vesicles. Sample concentration = 1 wt% in water.

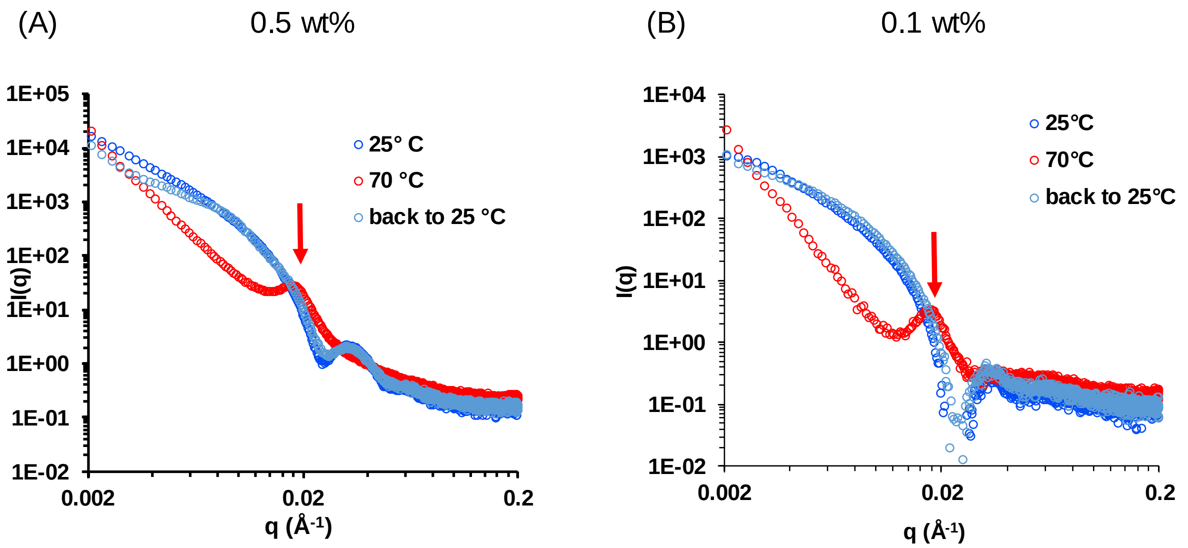

Figure S7. SAXS intensity (I) versus scattering vector (q) for PDMAm$_{36}$-*b*-PCMAm$_{360}$ (*sample 7*) in water at (A) 0.5 wt% and (B) 0.1 wt% at 25 °C (dark blue), 70 °C (red) and back to 25 °C (light blue). The arrows designate d, the reciprocal length corresponding to the inter-vesicle distance.

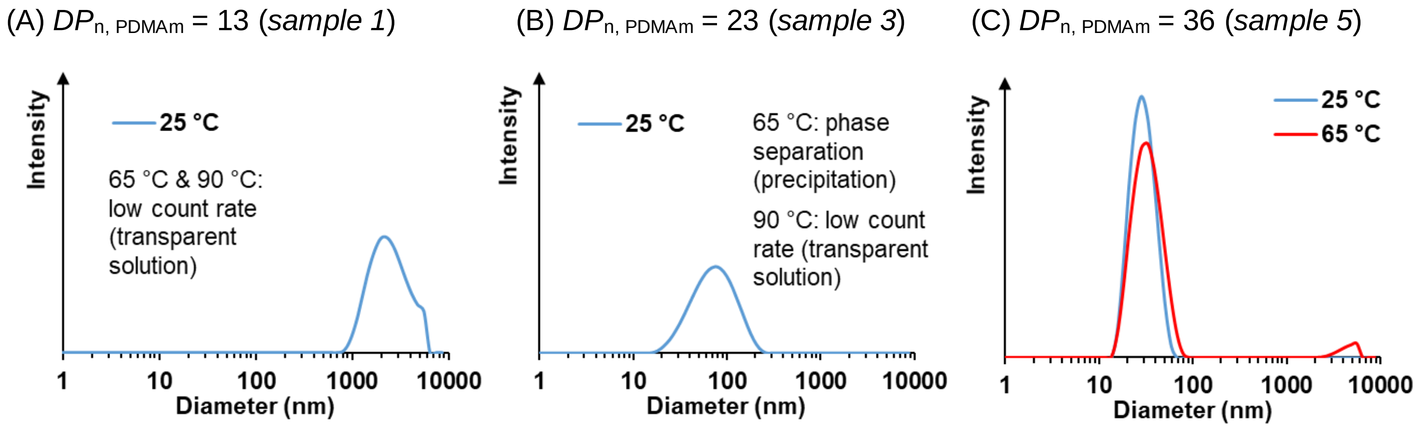

Figure S8. Size distributions determined by DLS of (A) PDMAm$_{13}$-*b*-PCMAm$_{\sim 90}$(*sample 1*), (B) PDMAm$_{23}$-*b*-PCMAm$_{90}$ (*sample 3*) and (C) PDMAm$_{36}$-*b*-PCMAm$_{91}$ (*sample 5*) dispersions prepared at 1 wt% in water at various temperatures.

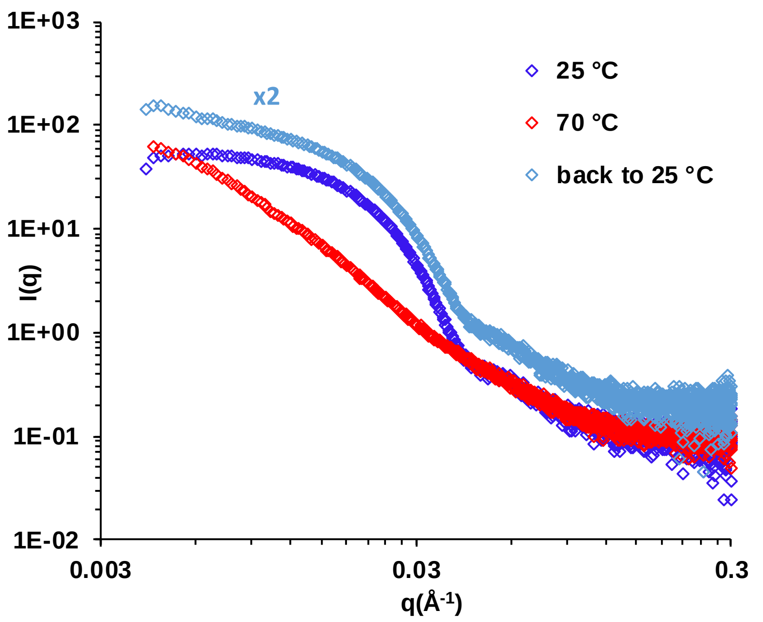

Figure S9. SAXS intensity (I) versus scattering vector (q) for $PDMAm_{36}$-*b*-$PCMAm_{91}$ (*sample 5*) in water at 0.1 wt% at 25 °C (dark blue), 70 °C (red) and back to 25 °C (light blue, intensity multiplied by 2 in order to visualize the signal, since it is a perfect overlay with the initial sample before heating).

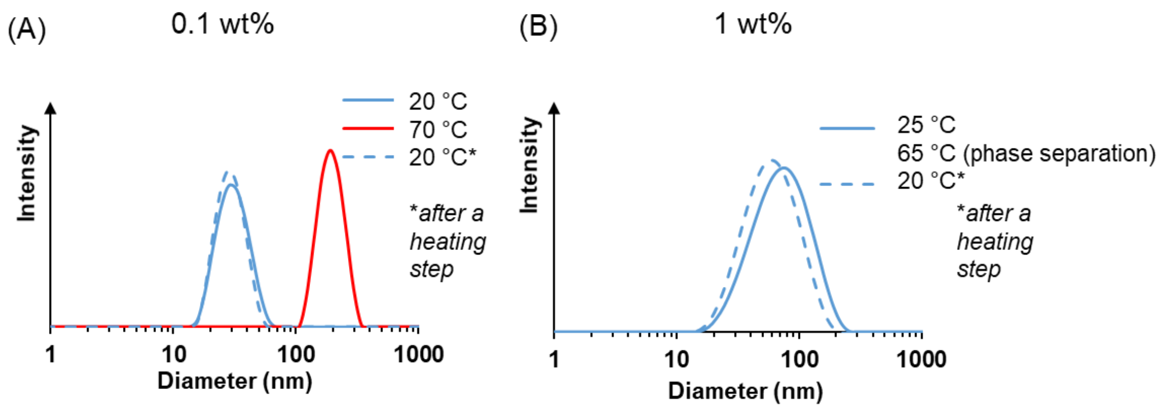

Figure S10. Size distribution of *sample 3* ($PDMAm_{23}$-*b*-$PCMAm_{93}$) at (A) 0.1 wt% and (B) 1 wt% in water determined by DLS at different temperatures. At 90 °C a very low scattering intensity was detected (kcps = 6 and 50, respectively for 0.1 wt% and 1 wt%).
